# Annotations

**Published:** 1909-04-24

**Authors:** 


### ANNOTATIONS.

### "The Hospital's" Commission on Beers.

Our readers will find in the present issue of The Hospital a Special Report on Beers, having reference to the mode of preparation and the chemical, physiological, dietetic, and medicinal properties of these essentially English beverages. Our Commissioners have spent a large amount of time and care upon their investigations into the scientific aspects of the subject, and the outcome of their researches occupies nearly twelve pages. It will be remembered that twice previously during the past three years we have devoted special supplements to reports upon alcoholic beverages, and the success of these analytical commissions, on whisky and light wines respectively, has led us to extend our inquiries to the no less interesting and important subject of Ales, Stouts, and Lager Beers. The more moderate amongst temperance reformers, with whose aspirations we desire to associate ourselves, have long recognised that the lighter forms of beer are, from the alcoholic standpoint, scarcely to be considered in the same category with ordinary strong liquors. None but the most rabid teetotallers regard a moderate consumption of light lager beer, for example, as injurious to the constitution or morals of the ordinary man. Moreover, whatever views they may hold upon the alcohol problem, and the position amongst alcoholic drinks which is to be accorded to the various kinds of beers, all will agree that nothing but good can come from the publication of a careful scientific and impartial investigation into the whole subject of the manufacture and medicinal properties of these beverages. Our readers will find in this report much that is of interest, and not a little that will be of practical value to them in the course of their professional duties. As we have remarked quite lately, the medical man has not only to tell his patients what they must not drink, but often also to advise them as to what they may. We feel confident that this report will have nothing but a beneficial influence upon the cause of true temperance.

### A Legal Analogy.

Some time ago a vigilant person, alleging that in these lax times the distinction between consultants and general practitioners is becoming effaced, proposed that the assumption of the former status should constitute as definite a step in the professional career as taking silk is to a barrister. It may be the anxious catching of the journalistic imagination, but this parallel is brought to mind by reports of the Prime Minister's speech at a recent meeting of a legal benevolent society. Mr. Asquith recalled the three methods of self-advancement recommended by tradition to the young barrister. They were—to write a text-book, to attend quarter sessions, or to marry an attorney's daughter. *Mutatis mutandis*, much the same advice might be given to members of the not vastly less crowded ranks of as yet obscure London medical consultants. In this, as in every higher walk of professional life, a prudent marriage counts for much; and although there is nothing in the metropolitan medical world exactly analogous to the second alternative—no polyclinic existing where case sheets are distributed like briefs for undefended criminals (a practice known in legal slang, we believe, by the single but expressive word "soup")—still, every aspirant to eminence in medicine needs no reminding of the wisdom of taking every chance of extending his clinical experience. The first-named plan is as popular in the medical profession as it ever was, and, we learn, still is, among the followers of Coke and Blackstone. As for text-books by young hospital clinicians, there is at present no evidence of a serious shortage in the output of this commodity.

### Oxygen Inhalations.

There are probably few of the practical applications of physiology to everyday affairs which have aroused the interest of the non-medical public to such an extent as has the administration of oxygen gas to athletes. The restoration of vigour and staying power to a man seriously exhausted by severe muscular efforts, such as those endured in running, boxing, and other strenuous games touches many questions of which the public is cognisant, particularly the ever-absorbing one of records. It is not, however, from the standpoint of whether or no the oxygenation of athletes is legitimate in sport that an article on this subject in the current issue of the *London Hospital Gazette* will chiefly interest the medical profession; but rather because of some of the technical details discussed. It appears that in the ordinary method of administering oxygen by a tube and funnel the percentage of this gas in the inspired air is raised only to 27 per cent. at the most. This was insufficient for the purposes of the particular experiments undertaken, but by a simple arrangement of a celluloid face-piece loosely tucked into the collar below and fastened by a cloth band round the head above, it was found not difficult to offer, with the same current from the cylinder, an oxygen percentage of 70. These facts and figures are well worthy of the attention of medical men, who so frequently have to supervise the management of the oxygen cylinder of a sick patient. Without entering at all into the statistical results of Dr. Leonard Hill's researches, the other point that may be alluded to is his conclusion that the fatigue which follows an athletic feat is mainly cardiac in origin, and due to want of oxygen. It will thus be seen that the London Hospital physiologist is not in agreement with Sir Lauder Brunton, to whose somewhat novel conception of fatigue toxins and antitoxins attention was drawn in our issue of April 10, 1909, on page 30 of the present volume.

